# Baseline selenium is associated with response to intravenous Methylprednisolone with selenium supplementation for thyroid eye disease in a selenium-sufficient area

**DOI:** 10.1038/s41598-025-29716-6

**Published:** 2025-11-24

**Authors:** Su Min Sung, Dong Cheol Lee

**Affiliations:** 1 Cheil Eye Hospital, 1 Ayang-ro, Dong-gu, Daegu, 41196 Republic of Korea; 2 Department of Medicine, Keimyung University Graduate School, 1095 Dalgubeol-daero, Dalseo-gu, Daegu, 42601 Republic of Korea; 3 Department of Ophthalmology, Keimyung University School of Medicine, 1035 Dalgubeol-Daero, Dalseo-Gu, Daegu, 42601 Republic of Korea

**Keywords:** Thyroid eye disease, Selenium, Clinical activity score, Intravenous steroid therapy, Biomarkers, Diseases, Endocrinology, Immunology, Medical research

## Abstract

Background Selenium (Se) status may influence activity and treatment response in thyroid eye disease (TED), but evidence from Se‑sufficient regions is limited. We aimed to evaluate the association between serum Se levels and clinical and serological outcomes in patients with TED treated with intravenous steroid therapy and Se supplementation. Serum Se increased from 125.05 to 167.75 µg/L throughout the follow‑up period (*p* < 0.001). Baseline Se correlated inversely with Clinical Activity Score (CAS) change at 2 months (ρ=−0.308; *p* = 0.047). A baseline Se threshold of 147.53 µg/L showed modest discrimination for ≥ 2-point CAS reduction (area under the curve [AUC] 0.653; sensitivity 50.0%; specificity 85.7%; positive predictive value 63.6%; negative predictive value 77.4%). Patients with Se ≥ 147.53 µg/L showed greater CAS reduction than those with lower levels (median − 2.0 vs. − 1.0, *p* = 0.012), and retained lower baseline‑adjusted final CAS in a rank‑based analysis of covariance (*p* = 0.017). Se levels were not significantly correlated with thyroid-stimulating hormone receptor antibody or thyroid-stimulating immunoglobulin changes. In a Se‑replete cohort receiving intravenous steroid plus Se, higher pretreatment Se was associated with greater CAS improvement. Given the modest AUC and limited power (59.5%), these hypothesis‑generating findings warrant prospective validation.

**Supplementary Information:**

The online version contains supplementary material available at 10.1038/s41598-025-29716-6.

## Introduction

Thyroid eye disease (TED) is an autoimmune condition characterised by orbital inflammation, tissue remodelling, and vision-threatening complications. Key contributors to its pathogenesis include oxidative stress and inflammatory pathways^1^. Selenium (Se), an essential trace element with antioxidant properties, has attracted interest as a potential modulator of disease activity^2^.

Notably, several studies have demonstrated that patients with TED have lower serum Se levels than those of healthy controls, and differences in serum Se levels are more pronounced in those with active or severe disease^3,4^. Reduced Se levels have been associated with elevated inflammatory markers, such as C–X–C motif chemokine ligand 10 (CXCL10), interleukin (IL)−23, and C–C motif chemokine ligand 2 (CCL2), and increased levels of thyroid autoantibodies^5,6^. However, the direct relationship between Se levels and clinical disease activity remains controversial, with inconsistent findings across studies^7^.

Se supplementation has shown promise in improving clinical outcomes in patients with mild TED, possibly by upregulating selenoproteins that modulate oxidative stress and inflammatory responses^8,9^. Therefore, the European Group on Graves’ Orbitopathy (EUGOGO) recommends Se supplementation for patients with mild TED in regions with low Se status^10^. However, the optimal timing of Se supplementation and its interaction with standard therapies, such as systemic corticosteroids, remain uncertain.

This study investigated the association between serum Se levels and clinical outcomes in patients with TED undergoing intravenous steroid therapy combined with Se supplementation. We evaluated the relationship between Se status and changes in Clinical Activity Score (CAS), thyroid-stimulating hormone receptor antibody (TSHR-Ab) levels, and thyroid-stimulating immunoglobulin (TSI) levels.

## RESULTS

### Baseline characteristics and longitudinal changes

**Fig. 1:**
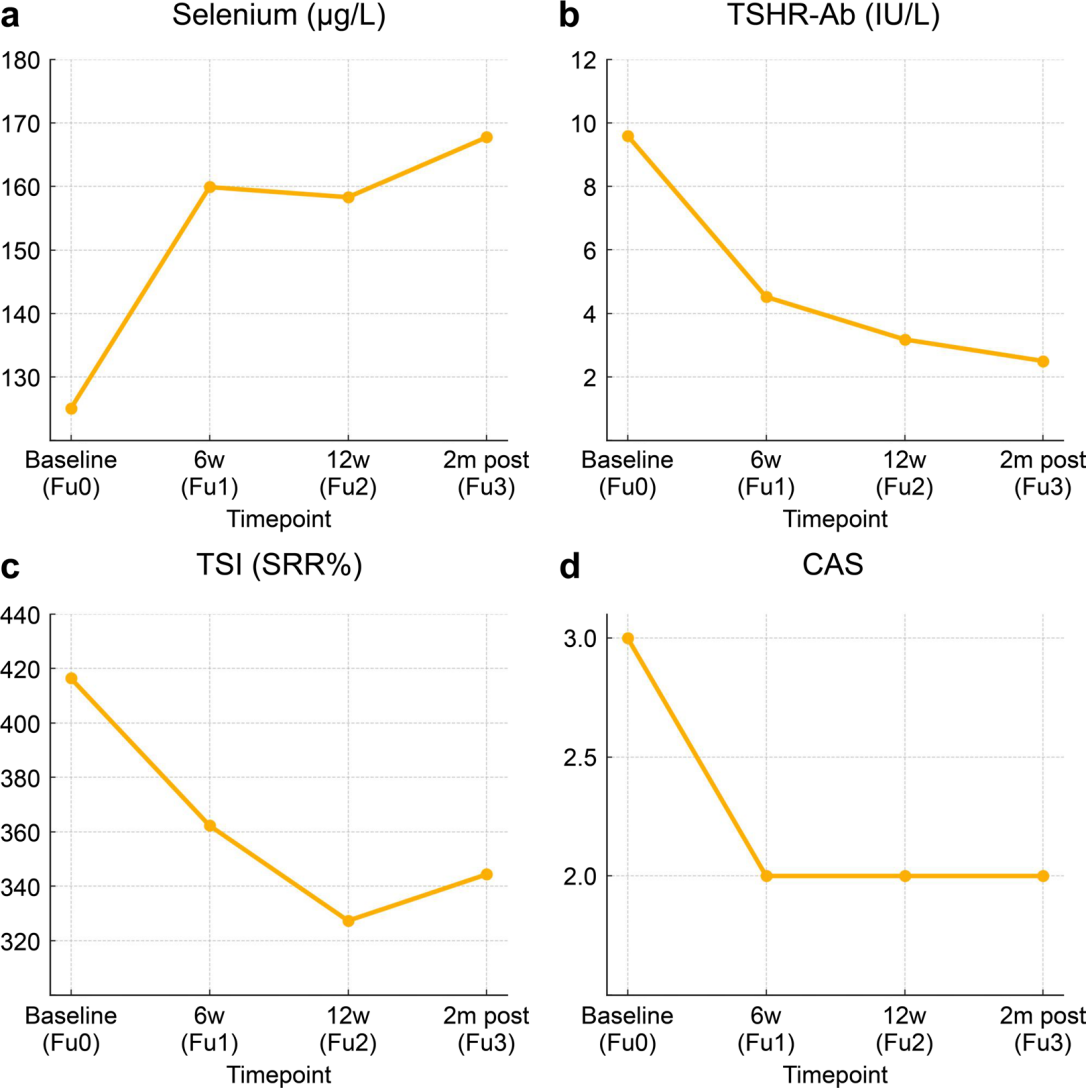
Changes in serum biomarkers and Clinical Activity Score (CAS) across follow-up time points. Line plots showing the mean values of (**A**) serum selenium (µg/L), (**B**) thyroid-stimulating hormone receptor antibody (TSHR-Ab, IU/L), (**C**) thyroid-stimulating immunoglobulin (TSI, SRR%), and (**D**) CAS across four visits: baseline (FU0), 6 weeks post-treatment (FU1), 12 weeks post-treatment (FU2), and 2 months after treatment completion (FU3).

The baseline demographic and clinical characteristics of the 42 enrolled patients are summarised in Table 1. No patient met the EUGOGO criteria for sight‑threatening TED. Of the 42 patients, 40 (95.2%) had moderate‑to‑severe disease and two (4.8%) had mild disease. All 42 eligible patients completed the full treatment and follow-up protocol and were included in the final analysis. At baseline (FU0), the median TSHR-Ab, mean TSI, and median CAS were 9.60 IU/L, 416.38 specimen‑to‑reference ratio (SRR%), and 3.00, respectively. These values significantly decreased to 2.50IU/L, 344.35 SRR%, and 2.00 (*p* < 0.001, *p* = 0.005, and *p* < 0.001, respectively) two months after intravenous steroid therapy (FU3) (Table 2; Fig. 1). Median Se increased from 125.05 to 159.90, 158.30, and 167.75 µg/L across FU0–FU3 (*p* < 0.001; Table 2; Fig. 1), while contemporaneous Se levels were not associated with TSHR‑Ab, TSI, or CAS at any time point (all *p* > 0.05). A meaningful clinical response (≥ 2‑point CAS reduction) was observed in 14 of the 42 patients (33.3%) at FU3. Among responders, the CAS items that improved most were eyelid injection and conjunctival injection (both *p* = 0.008), followed by pain on attempted gaze and eyelid swelling (both *p* = 0.031), whereas spontaneous pain, chemosis, and caruncle/plica swelling showed limited significance owing to low baseline prevalence (two‑sided exact McNemar test).

**Table 1:** Patients’ baseline demographic and clinical characteristics.

Characteristic	Value
Age (years)	40.95 ± 15.77
Sex (female/male)	29/13
Smoking Status (non-smoker/current smoker/former smoker)	32/8/2
History of diabetes mellitus (%)	11.90
Duration of TED symptoms (months)	2.00 [1.00; 4.00]
Thyroid functional status (hyperthyroid/euthyroid/hypothyroid)	38/3/1
Serum selenium (µg/L)	125.05 [108.43; 145.45]
TSHR-Ab (IU/L)	9.60 [4.21; 27.49]
TSI (SRR%)	416.38 ± 129.70
CAS	3.00 [2.00; 3.75]
Lid retraction OD (mm)	0.50 [0.00; 1.00]
Lid retraction OS (mm)	0.50 [0.00; 1.00]
Lid Lag (presence, %)	13.10
Lower scleral show (mm)	0.00 [0.00; 0.00]
Gaze limitation (presence, %)	19.05
Subjective diplopia (Bahn–Gorman scale)	0.00 [0.00; 0.75]
Exophthalmos OD (mm)	11.87 ± 2.05
Exophthalmos OS (mm)	12.51 ± 2.21
BCVA OD (logMAR)	0.09 ± 0.14
BCVA OS (logMAR)	0.10 ± 0.19
IOP OD (mmHg)*	17.85 ± 4.08
IOP OS (mmHg)*	17.95 ± 4.34

Values are presented as median [Q1; Q3] for non‑normally distributed variables, mean ± standard deviation for normally distributed variables, or number (%), unless otherwise indicated. Group comparisons were performed using the Mann–Whitney U or Fisher’s exact test as appropriate. Abbreviations: TED, thyroid eye disease; TSHR-Ab, thyroid-stimulating hormone receptor antibody; TSI, thyroid-stimulating immunoglobulin; CAS, clinical activity score; OD, ocular dexter; OS, ocular sinister; BCVA, best-corrected visual acuity; logMAR, logarithm of the minimum angle of resolution; IOP, intraocular pressure. Subjective diplopia was graded using the Bahn–Gorman scale (range 0–3).

*Baseline IOP data were missing in two patients; the mean baseline IOP was calculated from the data of the remaining 40 patients.

**Table 2:** Time-course changes in serum selenium, thyroid-stimulating hormone receptor antibody, thyroid-stimulating immunoglobulin, and clinical activity score (CAS).

Time point	Selenium (µg/L)	TSHR-Ab (IU/L)	TSI (SRR%)	CAS
FU0	125.05 [108.43; 145.45]	9.60 [4.21; 27.49]	416.38 ± 129.70	3.00 [2.00; 3.75]
FU1	159.90 [132.90; 180.93]	4.52 [2.12; 12.44]	362.30 ± 140.61	2.00 [1.00; 3.00]
FU2	158.30 [134.20; 194.53]	3.18 [1.27; 8.15]	327.30 ± 177.39	2.00 [1.00; 3.00]
FU3	167.75 [142.88; 235.75]	2.50 [1.14; 10.26]	344.35 ± 161.20	2.00 [1.00; 3.00]
p-value (test)	Friedman, *p* < 0.001	Friedman, *p* < 0.001	RM-ANOVA, *p* = 0.005	Friedman, *p* < 0.001

Values are presented as median [Q1; Q3] for non‑normally distributed variables and mean ± standard deviation for normally distributed variables. P-values are from Friedman tests for non-normally distributed variables (Se, TSHR-Ab, CAS) and repeated-measures ANOVA for normally distributed TSI. Abbreviations: CAS, clinical activity score; TSHR‑Ab, thyroid‑stimulating hormone receptor antibody; TSI, thyroid‑stimulating immunoglobulin; SRR%, specimen‑to‑reference ratio; FU, follow‑up visit; FU0, baseline; FU1, 6 weeks; FU2, 12 weeks; FU3, 2 months after treatment completion.

### Association between serum Se levels and CAS improvement (ΔCAS)

**Fig. 2:**
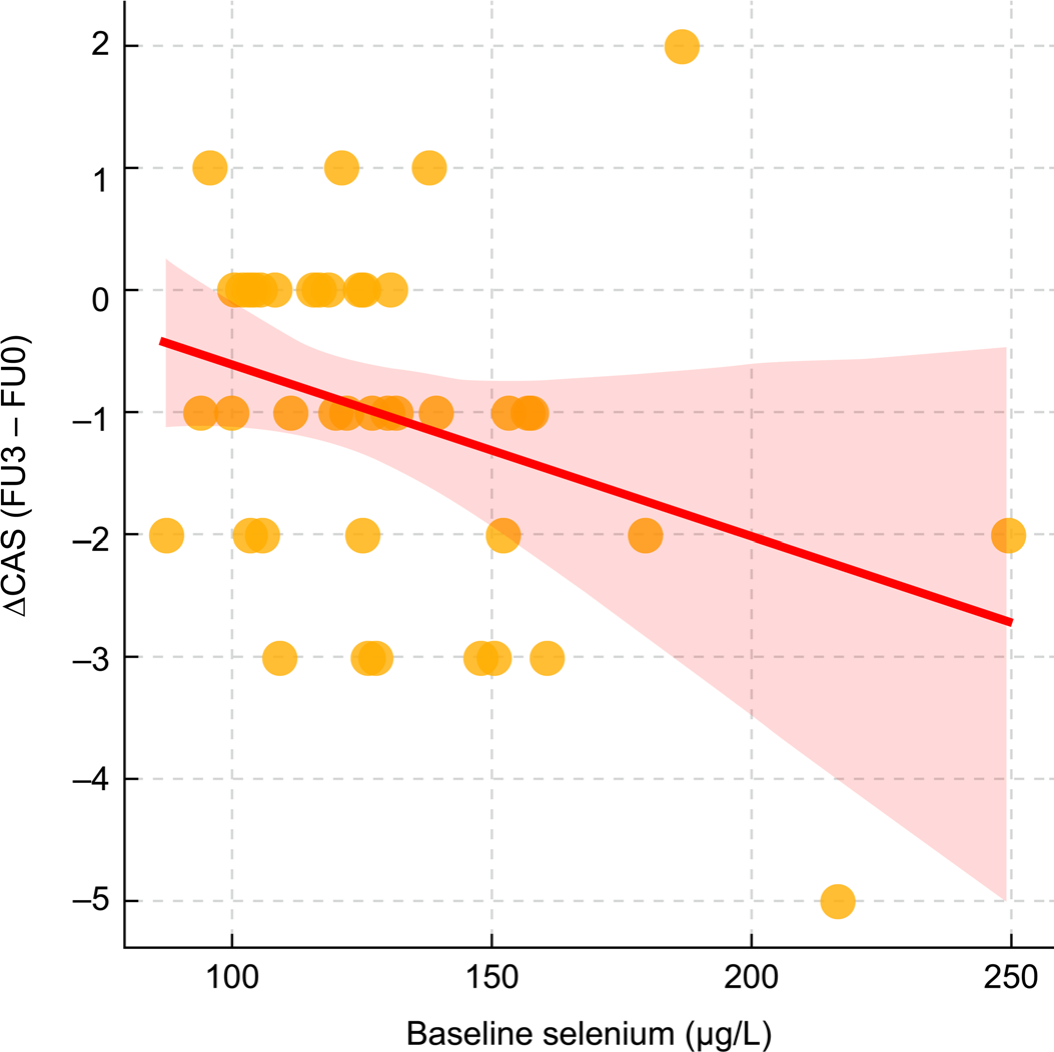
Correlation between baseline selenium level and change in Clinical Activity Score (CAS). A scatterplot showing the relationship between baseline serum selenium concentration (µg/L) and the change in CAS (ΔCAS) from baseline (FU0) to 2 months after treatment completion (FU3) in patients with thyroid eye disease receiving intravenous steroid therapy. A significant inverse correlation was observed (Spearman ρ = − 0.308, *p* = 0.047), suggesting that higher baseline selenium levels were associated with greater CAS improvement. A red loess curve was fitted to illustrate the trend.

**Fig. 3:**
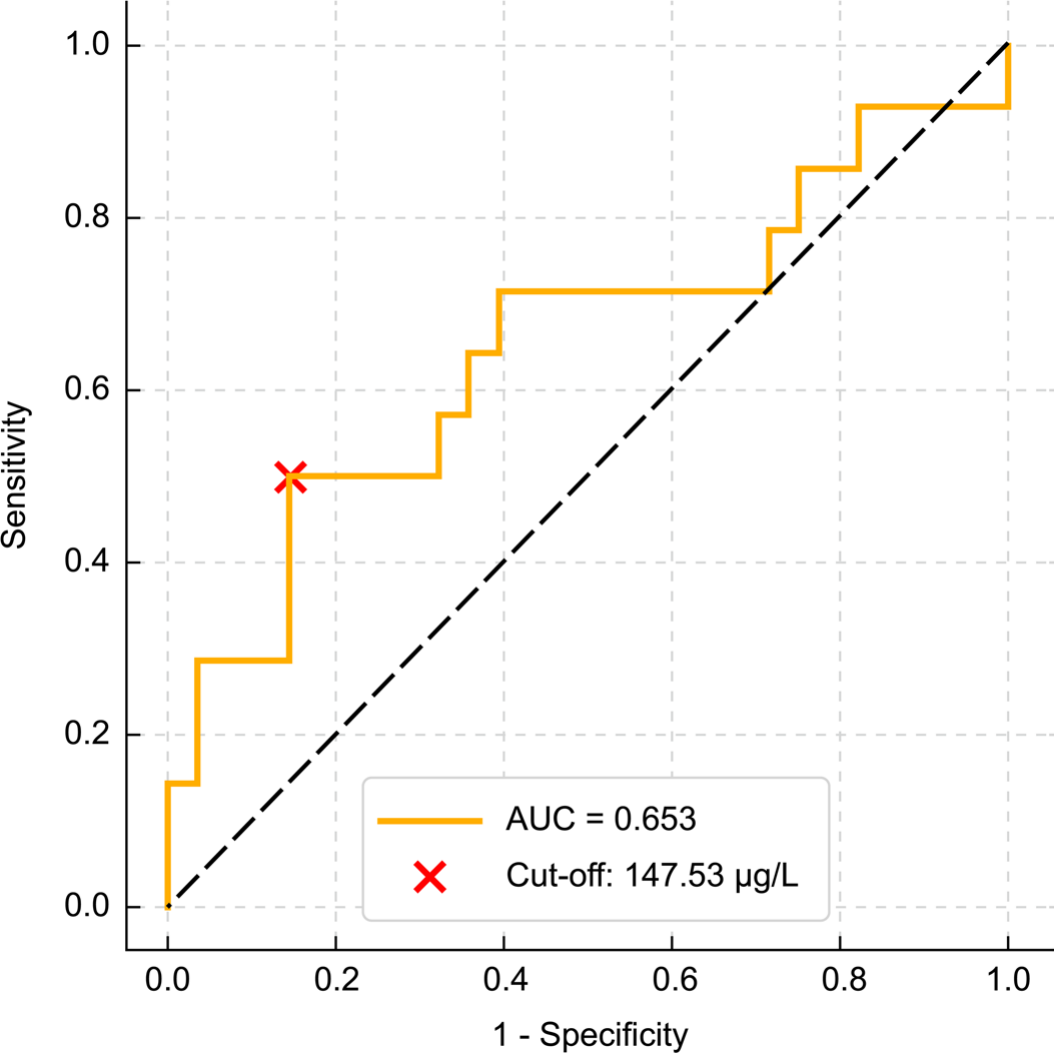
Receiver operating characteristic (ROC) curve evaluating baseline selenium as a predictor of Clinical Activity Score (CAS) improvement. An ROC curve was generated to assess the ability of baseline serum selenium level to predict meaningful clinical response, defined as a ≥ 2-point reduction in CAS. The optimal cutoff value was 147.53 µg/L, with an area under the curve (AUC), a sensitivity, and a specificity of 0.653, 50.0%, and 85.7%, respectively. The positive predictive value was 63.6% (35.4–84.8), and the negative predictive value was 77.4% (60.2–88.6).

**Fig. 4:**
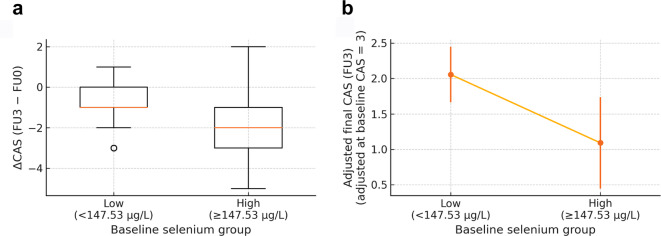
Comparison of the Clinical Activity Score (CAS) improvement and baseline-adjusted final CAS according to baseline selenium (Se) level. (**A**) Boxplot showing the change in CAS (ΔCAS) in patients with a baseline Se level of < 147.53 µg/L (*n* = 31) and ≥ 147.53 µg/L (*n* = 11). The box indicates the interquartile range (IQR), the horizontal line represents the median, and the whiskers represent 1.5×IQR. Patients with a Se level of ≥ 147.53 µg/L showed a significantly greater reduction in CAS than did those with lower levels (*p* = 0.012, Mann–Whitney U test). The median ΔCAS was − 2.0 in the high-Se group and − 1.0 in the low-Se group. (**B**) Estimated marginal means (EMMs) of final CAS (FU3) ± 95% CIs for each Se group derived from a linear analysis of covariance (ANCOVA) adjusting for baseline CAS. EMMs are displayed at baseline CAS = 3 (EMM high-Se group 1.09 vs. low-Se 2.06). In both the full cohort (*n* = 42) and the active-only subset (baseline CAS ≥ 3; *n* = 27), the high‑Se group had lower adjusted final CAS; *p* = 0.017 and *p* = 0.045, respectively (rank-based ANCOVA). FU0, baseline; FU3, 2 months after treatment completion.

Baseline Se showed an inverse correlation with ΔCAS (FU3–FU0) (Spearman ρ = −0.308; *p* = 0.047; Fig. 2). Receiver operating characteristic (ROC) analysis identified 147.53 µg/L as an exploratory threshold for predicting ≥ 2‑point CAS reduction (area under the curve [AUC] 0.653; sensitivity 50.0%; specificity 85.7%; Fig. 3). The positive predictive value (PPV) was 63.6%, and the negative predictive value (NPV) was 77.4%. Patients with a baseline Se level of ≥ 147.53 µg/L showed significantly greater CAS improvement than those with lower levels (median ΔCAS − 2.00 vs. −1.00; *p* = 0.012; Fig. 4A). No Se-related adverse effects were observed during the study period.

### Response‑stratified baseline comparison

At baseline, responders (≥ 2‑point CAS reduction, *n* = 14) had a higher CAS than non‑responders (median 4.00 vs. 2.50; *p* < 0.001) and were more likely to have Se ≥ 147.53 µg/L (50.0% vs. 14.3%; *p* = 0.024). Other baseline variables—including age, sex, TSHR‑Ab, TSI, duration of TED symptoms, smoking status, and thyroid function—were not significantly different (all *p* ≥ 0.05, Supplementary Table S1A).

### Se-stratified baseline comparison

When stratified by baseline Se (≥ 147.53 vs. < 147.53 µg/L), baseline CAS distributions were similar between the groups (median 3.0 [2.0–4.5] vs. 3.0 [2.0–3.0]; Mann–Whitney *p* = 0.533; Supplementary Table S1B). In the overall cohort, baseline Se was not associated with baseline CAS (Spearman ρ = 0.068; *p* = 0.669). By contrast, ΔCAS favoured the high‑Se group (median − 2.0 [− 3.0; −1.0] vs. − 1.0 [− 1.0; 0.0]; *p* = 0.012).

### Predictors of CAS improvement

In the univariable logistic regression, both higher baseline CAS (odds ratio [OR] 4.78; 95% CI 1.66–13.73; *p* = 0.004) and baseline Se ≥ 147.53 µg/L (OR 6.00; 1.35–26.60; *p* = 0.018) were associated with a ≥ 2‑point CAS reduction (Supplementary Table S2). In the multivariable model including baseline CAS, prior systemic glucocorticoid (GCS) exposure, and baseline Se status, high Se (≥ 147.53 µg/L) remained a significant independent factor associated with CAS improvement (aOR 13.84 [1.31–145.79]; *p* = 0.029; AUC 0.884, pseudo‑R² 0.41; Supplementary Table S3).

To mitigate bias from mathematical coupling between ΔCAS and baseline CAS, as well as regression‑to‑the‑mean, final CAS (FU3) was modelled as the dependent variable, with baseline CAS as the covariate in a rank-based analysis of covariance (ANCOVA). The effect of the Se group was significant (*p* = 0.017), with adjusted estimated marginal means (EMMs) of 1.09 (High-Se) vs. 2.06 (Low-Se) at baseline CAS = 3 (Fig. 4B; Supplementary Table S4).

### Active-only subset (baseline CAS ≥ 3; *n* = 27)

Results from the active-only subset were consistent with those of the overall cohort. Baseline Se again showed a significant inverse correlation with ΔCAS (Spearman ρ = −0.453; *p* = 0.018). Likewise, when the rank-based ANCOVA was repeated within this subset, the Se-group effect remained significant (*p* = 0.045; Supplementary Table S4), confirming that higher baseline Se was associated with greater improvement and a lower baseline-adjusted final CAS even when analyses were restricted to active cases.

### Clinical ophthalmic outcomes and their association with Se levels

Regarding clinical ophthalmic parameters, lid retraction decreased significantly from 0.50 [0.00; 1.00] mm at baseline to 0.25 [0.00; 0.50] mm at FU3 (*p* < 0.001). No significant correlations were found between baseline Se and baseline lid retraction, nor with its change to FU3 (*p* = 0.893, *p* = 0.489). The lid lag did not show any significant longitudinal change; however, the baseline lid lag was inversely associated with baseline Se levels (ρ = −0.331; *p* = 0.032). No significant associations were observed between baseline Se and lower scleral show (LSS) or gaze limitation.

Subjective diplopia scores improved significantly over time (*p* = 0.032); however, no correlation was found between Se levels and diplopia severity at any time point. No significant changes were observed in exophthalmos measurements across the study period (*p* = 0.065 for ocular dexter [OD], *p* = 0.054 for ocular sinister [OS], with a mean p-value of 0.120). However, the proportion of patients with an interocular proptosis difference of ≥ 2 mm significantly decreased (*p* = 0.012). Baseline Se correlated neither with baseline exophthalmos, nor with its change to FU3 (*p* = 0.709, *p* = 0.193).

## Discussion

In this Se‑replete cohort treated with intravenous methylprednisolone plus Se, higher pretreatment Se levels were associated with greater CAS improvement (ρ = −0.308, *p* = 0.047). An exploratory cut-off of 147.53 µg/L showed modest discrimination (AUC = 0.653). Rank-based ANCOVA further confirmed a lower adjusted final CAS in the high-Se group (*p* = 0.017). These findings suggest that baseline Se may serve as a pragmatic prognostic marker under combined therapy, although the limited sample size warrants cautious interpretation and external validation.

Although responders exhibited both higher baseline Se and higher baseline disease activity, this pattern does not indicate greater activity due to Se elevation at presentation. In the overall cohort, baseline Se was not associated with baseline CAS (ρ = 0.068; *p* = 0.669), and baseline CAS distributions were comparable between Se groups (median 3.0 in both; *p* = 0.533). By contrast, ΔCAS favoured the high‑Se group (median − 2.0 vs. − 1.0; *p* = 0.012). Therefore, the seemingly higher baseline CAS among responders likely reflects sampling variability and mathematical coupling, rather than a biological association between Se level and baseline disease activity.

In the multivariable model adjusted for baseline CAS and prior systemic glucocorticoid exposure, high Se (≥ 147.53 µg/L) remained a significant independent factor associated with CAS improvement. The wide confidence interval likely reflects the small sample size and limited events per variable in this exploratory model. Modelling final CAS with baseline adjustment addressed potential bias from regression‑to‑the‑mean and mathematical coupling, and the high-Se group maintained a significantly lower adjusted final CAS (*p* = 0.017). This consistent pattern, also evident in the active-only subset (baseline CAS ≥ 3; *n* = 27), supports an independent association between pretreatment Se status and therapeutic outcome.

In a European Thyroid Association consensus, 94% of members endorsed Se supplementation for TED^11^. Observational studies on Graves’ disease have shown that patients who achieved remission had higher Se levels (> 120 µg/L) than those who relapsed^12^, and a relatively insufficient Se level (≤ 93 µg/L) has been identified as an independent risk factor for severe TED^4^. Our finding that higher baseline Se levels predicted greater CAS improvement further supports a role for pretreatment Se status in modulating treatment response. Se supports key selenoproteins, glutathione peroxidase (GPx), and thioredoxin reductase (TrxR), that regulate thyroid redox balance^13,14^. By clearing reactive oxygen species/hydrogen peroxide generated during hormone synthesis, these enzymes reduce oxidative injury and oedema in orbital fibroblasts^14–16^. Thus, higher baseline Se may provide greater orbital antioxidant and anti-inflammatory reserve, improving treatment response. Concordantly, on‑treatment Se did not track contemporaneous activity, while pretreatment Se did, highlighting timing as a potential determinant of benefit.

However, no significant correlation was observed between Se levels and changes in TSHR-Ab or TSI. Recent findings suggest that Se protects the thyroid without directly influencing TSHR-Ab levels^17,18^. Because serial thyroid function data were not consistently available in our cohort, this mechanism could not be confirmed. Se may prevent localized damage to thyroid cells and orbital fibroblasts, reducing autoantigen release without directly suppressing autoantibody production^19^. Another study on Se reported delayed normalization of TSHR-Ab levels, implying that long-term studies are necessary to understand these complex relationships fully^20^.

Clinically significant improvement was observed in lid retraction and subjective diplopia following therapy. However, this improvement was not correlated with Se status. Baseline Se showed only a modest inverse association with lid lag, suggesting limited neuromuscular/fibrotic modulation. No significant change was observed in the exophthalmos values, although the values for patients with interocular differences of ≥ 2 mm decreased significantly. These patterns are consistent with prior findings indicating that Se-related benefits predominantly involve eyelid aperture and soft-tissue parameters, with minimal effects on proptosis or ocular motility^3,8,21^.

Generalisability is tempered by our Se-replete setting (median baseline 125.05 [108.43; 145.45] µg/L reference range), where supplementation effects may be smaller than in Se-deficient regions^22,23^. Se distribution and transport depend on selenoprotein P (SePP)^24^. Organic Se compounds, such as selenomethionine, can increase total serum Se through non-regulated incorporation into plasma proteins, often exceeding the threshold needed for optimal GPx (plateau > 100 µg/L) and SePP (110–125 µg/L) activity without further functional gain^22,25^. In autoimmune thyroid disorders, however, chronic oxidative stress and inflammation shifts Se utilisation toward immediate antioxidant defence (GPx/TrxR) and away from SePP, an effect amplified by IL-6–mediated suppression of hepatic SePP^26–28^. Optimising immune-related SePP may require higher serum Se (~ 146 µg/L)^13,29^, aligning with population data (The third National Health and Nutrition Examination Survey, NHANES III) associating 130–150 µg/L with the lowest risk of all-cause and cancer mortality^30^. Thus, individuals with Se levels below 122 µg/L could safely benefit from increasing their Se status to this optimal range^22,30^. The exploratory cutoff of 147.53 µg/L identified in our ROC analysis lies within this proposed optimal range, thereby reinforcing its clinical relevance. Prospective studies incorporating functional biomarkers (SePP concentration, GPx activity) are warranted to refine thresholds and clarify timing.

Se supplementation at 200 µg/day is generally considered safe; however, caution is warranted. Previous studies have suggested a U-shaped association between Se levels and health outcomes, indicating that Se supplementation may be most beneficial in individuals with suboptimal or deficient baseline levels^22^. A Se intake of > 400 µg/day is associated with selenosis, a toxicity syndrome characterised by symptoms such as gastrointestinal upset, fatigue, hair loss, and nail deformities^9,31^. No adverse events were observed in our cohort; however, prior clinical trials conducted in North America, where baseline Se levels were higher, reported increased risks of alopecia and dermatitis with long-term supplementation at the same dosage (200 µg/day of selenomethionine for 5.5 years)^32^. Therefore, assessing an individual’s Se status before supplementation and considering regional dietary Se intake are essential steps in optimising TED management.

In conclusion, pretreatment Se status was associated with greater CAS improvement after intravenous steroid therapy with Se supplementation in patients with TED. These findings are hypothesis-generating because causal inference is limited by the retrospective design, modest sample size (post-hoc power 59.5%), and absence of a non-Se control arm. Although documented Se supplementation within three months before baseline was excluded, unmeasured over-the-counter intake cannot be ruled out, potentially introducing residual confounding. Approximately one-third of participants had inactive TED (CAS < 3), adding heterogeneity despite consistent subgroup trends. Additional limitations include reliance on CAS without magnetic resonance imaging based inflammatory metrics, incomplete serial thyroid function data, and relatively short follow-up, all of which may restrict generalisability. Prospective randomised studies stratified by baseline Se sufficiency and incorporating functional biomarkers such as SePP and GPx are warranted to validate these findings and refine predictive thresholds and optimal timing for Se-guided management.

## Methods

### Ethics statement

This retrospective study was approved by the Institutional Review Board of Keimyung University Hospital (IRB No. 2024-01-063) and conducted in accordance with the 2013 (Fortaleza) revision of the Declaration of Helsinki. The requirement for informed consent was waived due to the retrospective nature of the study. However, written informed consent was obtained at the time of treatment initiation as part of the institutional protocol for intravenous steroid therapy.

### Study design and participants

This retrospective cohort study included 42 patients with TED treated between December 2020 and September 2023. All patients were treated under a standardised care protocol (EUGOGO guidelines) as part of routine clinical management. No patients were prospectively enrolled or followed for the purposes of this research, and all clinical data were retrospectively analysed after the completion of treatment and follow-up procedures.

We included patients aged ≥ 18 years who were diagnosed with TED according to EUGOGO criteria and had completed the full treatment protocol with adequate follow-up data. The exclusion criteria were age ≥ 80 years, prior orbital radiotherapy, orbital surgery within 6 months before enrolment, other orbital inflammatory diseases, or Se supplementation within 3 months before treatment. Prior systemic glucocorticoid exposure was abstracted and included as a covariate (one oral prednisolone taper in 2022; one intravenous methylprednisolone pulse course in 2020). The wash‑out intervals from the last prior exposure to baseline (FU0) were 188 and 693 days, respectively; the remaining 40 of the 42 patients (95.2%) were treatment‑naïve. This was not a matched cohort study, and no matching criteria were applied during participant selection.

### Treatment protocol

All patients received 4.5 g of intravenous methylprednisolone over 12 weeks, following the EUGOGO protocol (0.5 g weekly for 6 weeks, then 0.25 g weekly for 6 weeks). Se (200 µg/day, oral) was initiated at FU0 concurrently with the first infusion and continued through FU3 per institutional practice. According to EUGOGO guidelines, intravenous methylprednisolone is recommended for active moderate-to-severe TED (CAS ≥ 3). However, in this real-world cohort, 15 patients (35.7%) with inactive disease, including 13 moderate-to-severe and two mild cases, also received treatment at the ophthalmologist’s discretion based on patient‑specific considerations.

### Clinical and laboratory assessments

Baseline demographic and clinical data, including age, sex, smoking status, diabetes mellitus history, duration of TED symptoms, and thyroid functional status, were recorded. Comprehensive ophthalmic examinations were performed at baseline (FU0), 6 weeks (FU1), 12 weeks (FU2), and 2 months after treatment completion (FU3) by a single ophthalmologist. The clinical parameters monitored included lid retraction, lid lag, LSS, gaze limitation, subjective diplopia (Bahn–Gorman scale^33^, exophthalmos (measured using a Hertel exophthalmometer), best-corrected visual acuity, and intraocular pressure (IOP).

Blood samples were collected at each visit. Serum Se (µg/L), TSHR-Ab (IU/L), and TSI (SRR%) levels were measured using inductively coupled plasma mass spectrometry, electrochemiluminescence immunoassay, and a cell-based TSI reporter bioassay (Diagnostic Hybrids, Athens, OH, USA), respectively. Laboratory personnel were blinded to clinical information. Orbital magnetic resonance imaging was not part of routine care in this cohort. For bilateral measures (lid retraction, lid lag, LSS, exophthalmos, best-corrected visual acuity, IOP), patient‑level values were computed by averaging OD and OS at each visit.

### TED severity and activity criteria (EUGOGO)

Disease severity was classified per EUGOGO as mild, moderate‑to‑severe, and sight‑threatening. Sight‑threatening TED was defined by dysthyroid optic neuropathy and/or corneal breakdown (persistent epithelial defect/ulceration despite lubrication). Moderate‑to‑severe TED was defined by ≥ 1 of the following: lid retraction ≥ 2 mm, exophthalmos ≥ 3 mm above the sex-specific Korean upper limit of normal (predefined from published norms^34,35^, inconstant/constant diplopia (Bahn–Gorman 1–2/≥3), or ≥ moderate soft‑tissue involvement. Disease activity was evaluated using the 7-point CAS^36^, with scores of ≥ 3 indicating active disease.

### Outcomes

The primary outcomes were changes in serum Se levels over the treatment period; correlations between Se levels and TSHR-Ab, TSI levels, and CAS at each time point; and the association between baseline Se levels and CAS improvement at 2 months post-treatment.

### Statistical analysis

Statistical analyses were performed using SAS version 9.4 (SAS Institute, Cary, NC, USA) and Python version 3.11.8. Receiver operating characteristic (ROC) curve analysis and post hoc power calculation were conducted using Python’s scikit-learn (version 1.1.3), SciPy (version 1.9.3), and statsmodels (version 0.13.5) packages. Data normality was assessed using the Shapiro–Wilk test. Continuous variables are summarised as median [Q1; Q3] for non‑normally distributed data and as mean ± SD for normally distributed data. Longitudinal changes across time points were analysed using repeated-measures analysis of variance for normally distributed variables (TSI) and Friedman tests for non-normally distributed variables (serum Se, TSHR-Ab, and CAS). Friedman tests were also used for lid retraction, lid lag, LSS, gaze limitation, subjective diplopia, and exophthalmos. Paired binary changes in CAS components from FU0 to FU3 were assessed using McNemar’s exact test within responders.

Associations between baseline Se levels and changes in clinical parameters were evaluated using Spearman correlation analysis. A receiver operating characteristic (ROC) analysis was used to derive an optimal baseline Se threshold for predicting clinical response, defined a priori as a ≥ 2‑point decrease in CAS from FU0 to FU3. Baseline comparisons were performed for both response status (responders vs. non-responders) and Se status (Se ≥ 147.53 µg/L vs. < 147.53 µg/L, ROC-derived threshold) using the Mann–Whitney U test for continuous variables and Fisher’s exact test for categorical variables.

To explore potential confounders, univariate logistic regressions for age, sex, current smoking, thyroid status, duration of TED, TSHR-Ab, TSI, baseline CAS, and Se were performed. A multivariable model including baseline CAS and prior GCS exposure was additionally analysed to assess the independent effect of Se. A nonparametric rank-based ANCOVA was performed to test the effect of the Se group (≥ 147.53 vs. < 147.53 µg/L) on final CAS (FU3) with baseline CAS as a covariate. For interpretability, EMMs were additionally estimated using a linear ANCOVA at baseline CAS = 3, yielding adjusted final CAS values for each Se group. We prespecified an active-only subset (baseline CAS ≥ 3) to reduce heterogeneity from inactive cases and repeated both the Spearman correlation analysis between baseline Se and ΔCAS and the rank-based ANCOVA for final CAS within this subset.

A two-sided *p*-value of < 0.05 was considered statistically significant. Analyses focused on prespecified primary outcomes; multiplicity correction was not applied as secondary analyses were exploratory. No formal pre-study sample size calculation was performed. A post hoc power estimate for the ΔCAS comparison (using a nominal threshold of *p* < 0.05) was 59.5%, indicating the study was underpowered for definitive conclusions.

There were no missing data for clinical or serological outcomes across all follow-up time points. However, baseline IOP data were unavailable in two patients; therefore, the mean baseline IOP was calculated from the data of the remaining 40 patients.

## Supplementary Information

Below is the link to the electronic supplementary material.

Supplementary Material 1

## Data Availability

Data are available upon reasonable request from the corresponding author. Deidentified participant data used in this retrospective study are available from the corresponding author, Dongcheol Lee (tking33@naver.com), upon reasonable request. Data sharing is subject to approval by the Institutional Review Board of Keimyung University Dongsan Hospital and is permitted only for academic purposes. Additional documents, including the study protocol and statistical analysis plan, are available upon request from the corresponding author.
