# Supplementary material for: Baseline selenium is associated with response to intravenous Methylprednisolone with selenium supplementation for thyroid eye disease in a selenium-sufficient area
Source: Sci Rep. 2025 Nov 24;15:45276. doi: 10.1038/s41598-025-29716-6 (PMC12749133; doi:10.1038/s41598-025-29716-6)
Supplement: Supplementary file 1 — Supplementary Material 1 [file 41598_2025_29716_MOESM1_ESM.pdf]

Supplementary Information

Manuscript: "Baseline selenium is associated with response to intravenous methylprednisolone with selenium supplementation for thyroid eye disease in a selenium-sufficient area"

Su Min Sung, MD, Dong Cheol Lee, MD, PhD

Corresponding Author: Dong Cheol Lee, MD, PhD

All analyses were two-sided; statistical significance was defined as $p < 0.05$. All variables summarized as median [Q1; Q3] or n/N (%); p values from Mann–Whitney U or Fisher's exact test. Clinical response was defined as a ≥2-point reduction in Clinical Activity Score (ΔCAS) from baseline (FU0) to 2 months after treatment completion (FU3). High selenium was defined as ≥147.53 µg/L (receiver operating characteristic derived).

Supplementary Table S1A. Baseline characteristics by clinical response after intravenous methylprednisolone with selenium supplementation (responders vs non-responders)

Variable	Responders (n=14)	Non-responders (n=28)	p-value
Age (years)	32.50 [22.00, 46.25]	43.50 [33.75, 52.50]	0.138
Baseline CAS	4.00 [3.00, 4.00]	2.50 [2.00, 3.00]	0.0004
Baseline selenium (µg/L)	137.62 [113.10, 158.48]	121.62 [107.53, 130.56]	0.112
Baseline TSHR-Ab (IU/L)	5.33 [1.76, 10.62]	13.49 [5.73, 31.52]	0.060
Baseline TSI (SRR%)	456.25 [395.00, 534.20]	400.25 [320.18, 448.08]	0.070
Duration of TED symptoms (months)	3.75 [1.25, 7.75]	2.00 [0.88, 3.00]	0.058
Female, n/N (%)	10/14 (71.4%)	19/28 (67.9%)	1.000
High selenium ≥147.53 µg/L, n/N (%)	7/14 (50.0%)	4/28 (14.3%)	0.024
Current smoker, n/N (%)	1/14 (7.1%)	7/28 (25.0%)	0.233
Hyperthyroid status, n/N (%)	12/14 (85.7%)	26/28 (92.9%)	0.590
Prior GCS, n/N (%)	1/14 (7.1%)	1/28 (3.6%)	1.000

Abbreviations. CAS, Clinical Activity Score; TSHR-Ab, thyroid-stimulating hormone receptor antibody; TSI, thyroid-stimulating immunoglobulin; SRR%, specimen-to-reference ratio percentage; TED, thyroid eye disease; GCS, glucocorticoids.

Supplementary Table S1B. Baseline characteristics and change in Clinical Activity Score (ΔCAS) by baseline serum selenium status (≥ 147.53 µg/L vs < 147.53 µg/L)

Variable	Low Se (<147.53 µg/L) (n=31)	High Se (≥147.53 µg/L) (n=11)	p-value
Age (years)	38.00 [23.50; 50.50]	47.0 [36.50; 54.50]	0.203
Baseline CAS	3.00 [2.00; 3.00]	3.00 [2.00; 4.50]	0.533
Baseline TSHR-Ab (IU/L)	11.76 [4.71; 36.95]	6.35 [3.17; 13.49]	0.153
Baseline TSI (SRR%)	426.50 [333.15; 462.45]	436.50 [391.25; 501.35]	0.247
Duration of TED (months)	2.00 [1.00; 3.00]	3.50 [1.00; 7.50]	0.320
Female, n/N (%)	23/31 (74.2%)	6/11 (54.5%)	0.270
Hyperthyroid status, n/N (%)	29/31 (93.5%)	9/11 (81.8%)	0.277
ΔCAS (FU3 – FU0)	-1.0 [-1.0; 0.0]	-2.0 [-3.0; -1.0]	0.012

Abbreviations. CAS, Clinical Activity Score; TSHR-Ab, thyroid-stimulating hormone receptor antibody; TSI, thyroid-stimulating immunoglobulin; SRR%, specimen-to-reference ratio percentage; TED, thyroid eye disease; ΔCAS, change in CAS; FU3, 2 months after treatment completion; FU0, baseline.

Interpretation: Baseline characteristics were comparable between selenium groups. Despite similar baseline CAS distributions, ΔCAS favored the high-selenium group (-2.0 [-3.0; -1.0] vs -1.0 [-1.0; 0.0]; Mann–Whitney p = 0.012), consistent with the main analyses.

Supplementary Table S2. Univariable logistic regressions for the primary endpoint ≥2-point reduction in Clinical Activity Score (exploratory covariate screening)

Variable	OR	CI_low	CI_high	p-value
Baseline CAS	4.78	1.66	13.73	0.004
High selenium (≥147.53 μg/L)	6.00	1.35	26.60	0.018
Selenium per 10 μg/L	1.25	0.99	1.58	0.058
TSI (SRR%)	1.01	1.00	1.01	0.059
Duration of TED symptoms (months)	1.11	0.97	1.27	0.142
Age (years)	0.97	0.93	1.01	0.184
Current smoker (yes vs no)	0.23	0.03	2.10	0.193
TSHR-Ab (IU/L)	1.00	0.98	1.01	0.613
Hyperthyroid status (yes vs no)	0.46	0.06	3.68	0.465
Female (vs male)	1.18	0.29	4.83	0.814

"OR" denotes odds ratio; "CI_low/CI_high" denote the lower/upper bounds of the confidence interval. "Selenium per 10 μg/L" is the OR per 10-μg/L increase in serum selenium.

Abbreviations. CAS, Clinical Activity Score; TSI, thyroid-stimulating immunoglobulin; SRR%, specimen-to-reference ratio percentage; TED, thyroid eye disease; TSHR-Ab, thyroid-stimulating hormone receptor antibody.

Supplementary Table S3A. Prior systemic glucocorticoid exposure by clinical response

Metric	Value
Responders with prior systemic glucocorticoid	1
Non-responders with prior systemic glucocorticoid	1
Fisher's exact p	1.000

Supplementary Table S3B. Multivariable logistic regression adjusted for baseline Clinical Activity Score and prior systemic glucocorticoid

Variable	OR	CI_low	CI_high	p-value
High selenium ≥147.53 μg/L	13.84	1.31	145.79	0.029
Baseline CAS	6.40	1.71	23.97	0.006
Prior GCS	0.44	0.00	26810.72	0.885

Model performance: AUC=0.884; McFadden pseudo-R^2=0.410. All predictors (selenium ≥ 147.53 μg/L, baseline CAS, prior GCS) were entered simultaneously (forced-entry).

"OR" denotes odds ratio; "CI_low/CI_high" denote the lower/upper bounds of the confidence interval. Se≥147.53 μg/L threshold was ROC-derived. Abbreviations. CAS, Clinical Activity Score; GCS, glucocorticoids; AUC, area under the ROC curve; ROC, receiver operating characteristic.

Supplementary Table S4. Rank-based analysis of covariance of final Clinical Activity Score adjusted for baseline score, with estimated marginal means at baseline score 3

Subset	EMM High-Se (at baseline CAS = 3)	EMM Low-Se (at baseline CAS = 3)	p-value
Full cohort (n = 42)	1.09 [0.45–1.74]	2.06 [1.66–2.45]	0.017
Baseline-active subset (CAS ≥3; n = 27)	0.79 [−0.35–1.92]	2.08 [1.50–2.65]	0.045

Reported for the full cohort (n = 42) and baseline-active subset (CAS ≥ 3; n = 27).

Nonparametric rank-based ANCOVA was used to mitigate change-score coupling and regression-to-the-mean. For interpretability, estimated marginal means (EMMs) were additionally estimated using a linear ANCOVA at baseline CAS = 3, yielding adjusted final CAS values for each selenium group, with each EMM followed by its corresponding 95% confidence interval in parentheses. Abbreviations. CAS, Clinical Activity Score; Se, selenium.
